# A Shoot Phenological Study of Certain *Phyllostachys* Bamboo Taxa Under Central European Climatic Conditions

**DOI:** 10.3390/plants13243592

**Published:** 2024-12-23

**Authors:** Khin Nyein Chan, Anikó Veres, Zhiwei Liang, Szilvia Kisvarga, András Neményi

**Affiliations:** 1 Institute of Genetics and Biotechnology, Hungarian University of Agriculture and Life Sciences (MATE), 2100 Gödöllő, Hungary; chan.khin.nyein.2@phd.uni-mate.hu (K.N.C.); veres.aniko@uni-mate.hu (A.V.); 2 Institute of Agronomy, Hungarian University of Agriculture and Life Sciences (MATE), 2100 Gödöllő, Hungary; zhiwei.liang@phd.uni-mate.hu; 3 Institute of Landscape Architecture, Urban Planning and Garden Art, Hungarian University of Agriculture and Life Sciences (MATE), 2100 Gödöllő, Hungary; kisvarga.szilvia@uni-mate.hu

**Keywords:** *Phyllostachys*, shoot phenology, air temperature, soil temperature

## Abstract

This study aimed to understand the phenological changes in the shoots of temperate bamboo *Phyllostachys* spp. grown in Hungary, with a focus on how these changes were influenced by local climatic conditions. Data collected over two years on shoot phenology were analyzed with weather variables, especially air temperature and soil temperature. Shoot emergence date, shoot numbers, and shooting period were recorded within and between *Phyllostachys* spp. The date of shooting was observed between May and July, and species started shooting in May the most, followed by June. Only one species, *P. sulphurea*, started shoot emergence in July. Shoot emergence started earlier in 2022 than in 2023 and ranged from 3 days to 27 days. Bamboo shoot phenology was strongly influenced by the air temperature and soil temperature each year. According to our regression analysis, the dates of shoot emergence were influenced by air temperature (r^2^ = 0.819) in 2022 and 2023 (r^2^ = 0.781), and soil temperature also influenced shoot emergence (r^2^ = 0.956) in 2022 and 2023 (r^2^ = 0.769). Sharp air temperature changes between the shooting season and the period before shooting were considered as the reasons for early shooting. The soil temperature in April before the start of shoot emergence was 8.15 °C, increasing to 14.1 °C during shooting time in May. Abrupt fluctuations of air temperature between the shoot emergence season and the month prior to shooting commencement determined early shooting or late shooting. The soil temperature had to reach 9 °C or above prior to shooting time, as this was found to be a critical temperature for shoot emergence in *Phyllostachys* bamboo taxa.

## 1. Introduction

Bamboo is becoming popular around the world because of its versatile uses; it is important both economically as well as environmentally. It is regarded as an alternative to timber because it is a potential sustainable resource in the construction industry [1] and as a potential plant for providing solutions to climate change mitigation. A 10% increase in the global forest area from 2015 to 2050 may lead to the sequestration of atmospheric CO_2_, which will decrease the CO_2_ concentration in the atmosphere by 12% [2]. Bamboo could be a candidate plant for climate change mitigation because of its fast-growing nature and high carbon sequestration [3], i.e., 50% higher than that of Chinese fir, a fast-growing forest tree [4]. Moso bamboo (*Phyllostachys* spp.) was reported to have a high potential for carbon sequestration [5,6], as it could be harvested with 6.0 Mg C ha^−1^ to 7.6 Mg C ha^−1^ annually [6]. The global area covered by bamboo increased from about 22 million ha in 1990 to 35 million ha in 2020 [7]. According to FAO [7], bamboo cultivation or forests were recorded to cover 46,480,000 ha in Africa, 248,770,000 ha in Asia, 1,250,000 ha in the Caribbean, and 53,890,000 ha in South America. The majority of bamboo genera and species originate from Asia and have been utilized for thousands of years; however, the first bamboos were only introduced to Europe in the 19th century as ornamentals [8]. The introduction of new species into European ornamental horticulture has been continuous ever since. *Phyllostachys* spp. were among the first taxa to be introduced into Europe: *P. aurea* to Germany in 1977/78, *P. congesta* to Germany and France in 1981, *P. arcana* to Germany in 1991, etc. Now, there are some botanical gardens containing bamboo collections in Europe too, especially in Belgium, Germany, Italy, France, the Netherlands, and Spain [9].

Plant phenology is the study of the timing of the phases in the plant life cycle from the beginning, i.e., germination, to the end, e.g., harvesting time [10]. Studying plant phenology is of great importance, because it can provide valuable information about the relationship between plants and the environment [11]. It can give us some benefits for studying climate change and biodiversity, understanding ecosystem functions, and improving crop management. Changes in plant phenology, such as shoot phenology, are directly connected to climate change [12]. Plants are immobile living organisms that often endure adverse weather conditions. In order to adapt to the prevailing climate, they undergo changes in their mechanisms and behavior. Mutation is one of the reasons plants autonomously adjust their mechanisms to cope with varying weather conditions. Additionally, they modify their phenological stages in response to the local climate they encounter. Even a 1 °C increase in early spring can change the growing season by initiating it 7 days earlier in some woody plants [13]. The temperature response of perennial plants is greater than that observed in annual agricultural crops [14]. The phenology changes of 78 cultivated crops were examined in Germany; it was noted that their events occurred significantly earlier than 53 years ago [14]. Therefore, plant phenology is important for studying and understanding plant responses to the environmental changes that influence the ecosystem, biodiversity, and agriculture.

Phenological data are crucially important for providing information to track changes in vegetation over time, particularly for observing the phenological stages of bamboo forests, such as shoot emergence and adaptability in the study area. In addition, these data can help in assessing the impact of environmental factors on bamboo growth, contributing to a better understanding of how climate change may affect bamboo species and their genetic diversity. By integrating remote sensing techniques with these environmental datasets, researchers can gain insights into the spatial and temporal dynamics of bamboo ecosystems, ultimately supporting conservation efforts and sustainable management practices [15].

Regarding bamboo plants, there is wide diversity around the world. Depending on the species, there is a specific period for shoot emergence and the culm height growth rate. Bamboo has a long life cycle, and flowering occurs after 30–120 years of growth. So, reproduction from seeds is a rare event; therefore, the annual new culm development is a critical factor for the vegetative growth of bamboo plants, which depends on shoots arising from the rhizomes every year. The shoot phenology of different bamboo species has mostly been studied in China [16,17,18,19,20,21,22,23], but the majority of research centers around the most economically important species, *Phyllostachys edulis* (syn. *P. pubescens*), or moso bamboo [16]. Most *Phyllostachys* spp. have shoot emergence from March to May–June in their native Chinese habitat [17,22,23]. In Europe, Gratani, L. et al. [24] observed the shoot emergence of three *Phyllostachys* spp. in April and May at the Botanical Garden of Rome. The shoot phenology of the observed three bamboo taxa was different in the two countries under different climatic conditions. Therefore, the determination of the shooting period of *Phyllostachys* spp. (that have already been introduced to Europe) is needed in order to study their adaptability and phenological and environmental responses.

The purposes of this research were (a) to observe and record the shoot phenology between and within some *Phyllostachys* spp., (b) to study the effect of environmental parameters, namely air and soil temperature, on the shoot phenology of *Phyllostachys* spp., and (c) to compare the differences in the observed shoot phenology of *Phyllostachys* spp. based on differences in environmental parameters during the two observation years.

## 2. Results

### 2.1. Descriptive Statistics of Shoot Phenology of Bamboo Genotypes

#### 2.1.1. Data Analysis of Shoot Phenology Collected in 2022

The shoot phenology of bamboo genotypes was analyzed using descriptive and *t* tests. All data showed significant differences in both years of observation (Table 1 and Table 2). Most *Phyllostachys* species produced new shoots from May to July of each year. A total of 32 *Phyllostachys* taxa produced new shoots from May and finished shooting in June in 2022. The earliest date of shoot emergence was found in *P. aureosulcata* f. *harbin inversa* (2 May) and the latest shoot emergence was found in *P. sulphurea* f. *viridis* and *P. sulphurea* f. robert young (4 July) in 2022. The number of new shoots varied in different bamboo genotypes. The maximum total number of new shoots was observed in the medium sized taxa, such as *P. manni* (60), followed by *P. viridiglaucescens* (57), *P. yunhoensis* (53), and *P. aureosulcata* (51), respectively, while few new shoots emerged in some larger sized taxa such as *P. iridescens*, *P. vivax* f. *huangwenzhu*, *P. sulphurea* f. *houzeauana*, *P. sulphurea* f. *viridis*, and *P. sulphurea* f. rober young. Twelve out of 42 bamboo genotypes showed lower survival rates of new shoots (less than 50%), while 11 genotypes showed survival of all new shoots. The longest shooting period was found in *P. bambusoides* f. *holochrysa* (49 days), followed by *P. viridiglaucescens* (38 days), *P. yunhoensis*, *P. prominens*, and *P. aureosulcta* f. *harbin inversa* (31 days).

#### 2.1.2. Data Analysis of Shoot Phenology Collected in 2023

Regarding shoot phenological data collected in 2023, there was significant difference between all bamboo genotypes (Table 2). With regards to the date of shoot emergence in 2023, in 23 out of 42 bamboo genotypes, shoot emergence was observed in May. Among those 23 genotypes, only two taxa, *P. aureosulcata* f. *harbin inversa* and *P. arcana* f. *luteosulcata*, were found to have the earliest shoot emergence on the 2nd of May and 5th of May, respectively, while the other 21 taxa produced new shoots from the 3rd week of May. The last date of new shoot emergence was found to be on the 14th of July. The maximum number of shoots was observed in *P. flexuosa* (60), followed by *P. rutila* (45) and (35) in *P. aureosulcata*, *P. aureosulcata* f. *harbin inversa*, and *P. aureosulcata* f. *pekinensis*. The lowest number of shoots was found in 4 taxa, namely, *P. nigra* var. *nigra*, *P. vivax* f. *huangwenzhu*, *P. viridis* f. *houzeauana*, and *P. sulphurea* f. *viridis*. Seventeen of the studied bamboo genotypes had low survival rates of new shoots (less than 50%), while 12 genotypes had 100% survival rate of new shoots. The longest shooting period was observed to be 28 days in *P. glauca*, followed by *P. angusta* with a 25 day long shooting period.

#### 2.1.3. Descriptive Statistics of Shoot Phenology in 2022 and 2023

Table 3 shows the descriptive analysis of shoot phenological data collected in the two observation years. According to a data analysis of the two years’ data, a high CV % was observed, which means that high variability in shoot phenology was observed between the bamboo genotypes used in this study in both years.

### 2.2. Comparison of the Shoot Phenological Changes Between the Two Observation Years

**Figure 1:**
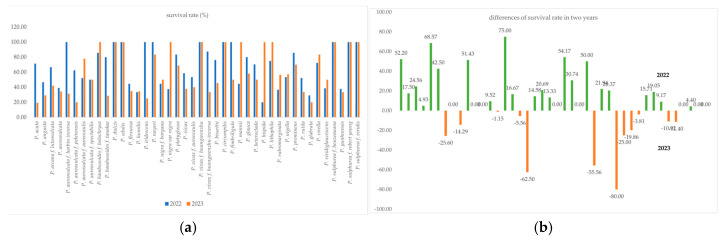
Comparison of the survival rate of new shoots of bamboo taxa between the two years (**a**,**b**).

The survival rate of new shoots in the bamboo genotypes during the two observation years is shown in Figure 1. Most bamboo genotypes showed a higher new shoot survival rate in 2022 than in 2023 (Figure 1b). Interestingly, *P. dulcis*, *P. edulis*, *P.vivax* f. *huangwenzhu*, *P. circumpilis*, *P. sulphurea* f. *houzeauana*, *P. sulphurea* f. robert young, and *P. sulphurea* f. *viridis* showed consistent survival rates in both years (Figure 1b).

**Figure 2:**
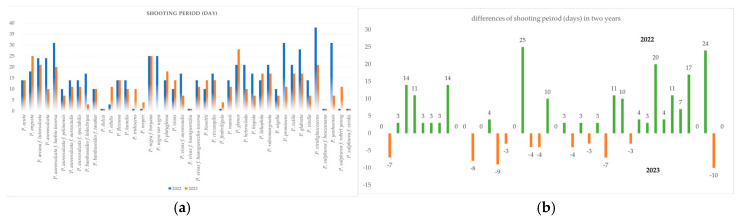
Comparison of shooting period of bamboo genotypes in the two years (**a**,**b**).

Differences in the length of the shooting period during the two observation years in 42 bamboo genotypes are shown in Figure 2a,b. The length of the shooting period was longer in 2022 than in 2023 in some bamboo genotypes. Nine taxa, namely, *P. acuta*, *P. bambusoides* f. *tanakae*, *P. dulcis*, *P. flexuosa*, *P. nigra* f. *boryana*, *P. vivax* f. *huangwenzhu*, *P. viridis* f. *houzeauana*, and *P. sulphurea* f. *viridis* produced the same length shooting period in both years. Except for *P. acuta*, *P. bambusoides* f. *tanakae*, *P. flexuosa*, and *P. nigra* f. *boryana*, the other five species produced only a single day of shooting in both years. *P. nigra* var. *nigra*, *P. yunhoensis*, and *P. prominens* also showed large differences in the lengths of the shooting periods (25, 24, and 20 days) during the two years.

**Figure 3:**
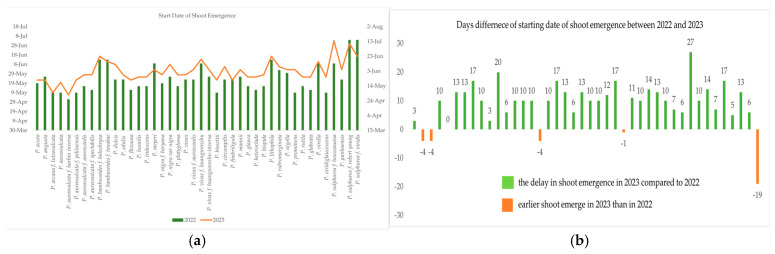
(**a**,**b**) Date of first new shoot emergence of bamboo genotypes during the two observation years.

**Figure 4:**
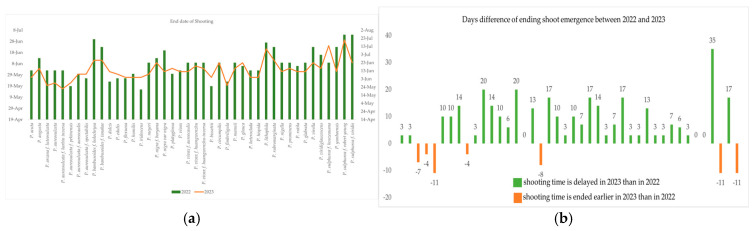
(**a**,**b**) Difference of the last date of shoot emergence of bamboo genotypes in the two-year study period.

The first and the last dates of shoot emergence in the different bamboo genotypes during the two observation years are shown in Figure 3a,b and Figure 4a,b. Figure 3a,b shows the difference in the starting date of shoot emergence in the different bamboo genotypes during the two observation years. In *P. aureosulcata* f. *harbin inversa*, there was no change in the date of new shoot emergence in both years. Compared to 2022, there was a delay in shoot emergence in most of the taxa in 2023. Most taxa showed a delay of more than 10 days in shoot emergence. Compared to 2022, *P. sulphurea* f. *viridis* showed a 19 day delay in shoot emergence in 2023, while in the other taxa, there were between 1 and 4 days of delays in shoot emergence. In our study, only five taxa showed earlier shoot emergence in 2023 compared to 2022. 

The last dates of shoot emergence in bamboo genotypes in the two observation years are shown in Figure 4a,b. As expected, most of the species ended shooting later in 2023 compared to 2022. The difference of last shooting date in days ranged from 3 days to 35 days in 2023 compared to 2022. Some bamboo genotypes, namely, *P. meyeri*, *P. virella*, and *P. viridiglaucescens*, produced the same date of last shoot emergence in both years in our study. *P. bambusoides* f. *holochrysa* showed an earlier last shooting date in 2023 compared to 2022. Another six *Phyllostachys* genotypes showed earlier last dates of shooting, i.e., between 4 days to 11 days earlier in 2023 compared to 2022.

#### Air Temperature, Soil Temperature, Relative Humidity and Precipitation During Shooting Season in the Year of 2022 and 2023

Weather parameters before and during the shooting period were collected in 2022 and 2023 (Table 4). Average air temperature/month, average air maximum temperature/month, average minimum air temperature/month, soil temperature, soil water content, precipitation, and relative humidity were collected in March and April before the observations started in both years. Air temperature and soil temperature were found to be higher in 2022 than in 2023; also, the soil water content was higher in 2023 than in 2022. Relative humidity was similar in both years. Inversely, during the observations, air temperature (daily and average), maximum (daily and average) and minimum temperature (daily and average), and soil temperature were higher in 2022 than in 2023 during the shooting period. However, the precipitation and relative humidity and soil water content were higher in 2023 than in 2022.

### 2.3. Correlation Analysis of Bamboo Phenology and Environmental Parameters

A correlation analysis was carried out to study the relationship between environmental parameters (Table 5) and bamboo shoot phenology. Table 6 shows the correlation between environmental parameters and shoot phenology during the two years of observation. During the analysis for 2022 (Table 6), the date of shoot emergence (DOE) showed positive correlations with air temperature (0.883**), maximum air temperature (0.882**) and minimum air temperature (0.881**), soil temperature (0.978*), and precipitation (0.413*), while it was negatively correlated with soil water content (−0.998**). No correlation occurred between DOE and relative humidity in the 2022 shooting season. Comparing the total number of shoots (TOS) with environmental parameters, only soil water content (WC50) correlated positively (0.367*), and soil temperature (ST50) was negatively correlated with TOS (−0.381*). The number of surviving shoots (NOL) only showed a positive correlation with WC50 (0.40**), while there was a negative correlation with soil temperature (−0.436**). The survival rate (SR) was determined to be positively correlated with all data for air temperature and soil temperature, while it was negatively corelated with soil water content (−0.404**). The shooting period (SHP) correlated with only two parameters: positively with soil water content (0.373*), and negatively with soil temperature (−0.422**).

According to the results for 2023 (Table 6), the date of shoot emergence (DOE) correlated positively with average air temperature, average maximum air, and minimum air temperature (0.569**, 0.575**, 0.577**). The total number of shoots (TOS) was negatively correlated with soil temperature; however, no correlation with air temperature was observed. Total number of shoots (TOS) and the number of surviving shoots were negatively correlated with average air temperature (−0.449**, −0.424**), maximum air temperature (−0.446**, −0.421**), minimum air temperature (−0.445**, −0.420**), and soil temperature (−0.582**, −0.471**) but were positively correlated with soil water content (WC50) (0.590**, 0.480**). The survival rate (SR) was positively correlated with all air temperature and soil temperature parameters (0.381*, 0.378*, 0.377*, 0.670**) in our study and negatively correlated with soil water content (−0.628**). Similarly, shooting period (SHP) was also observed to be in negative correlation with average air temperature (−0.509**), average maximum air temperature (−0.507**), average minimum air temperature (−0.506**), and soil temperature (−0.472**), while there was a positive relationship with soil water content (0.507**).

### 2.4. Influence of Environmental Parameters on Bamboo Phenology

**Figure 5:**
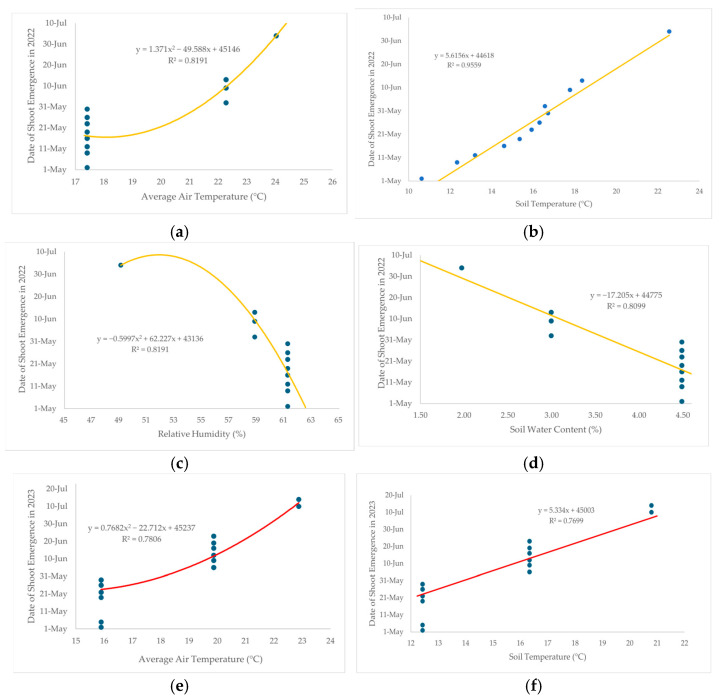

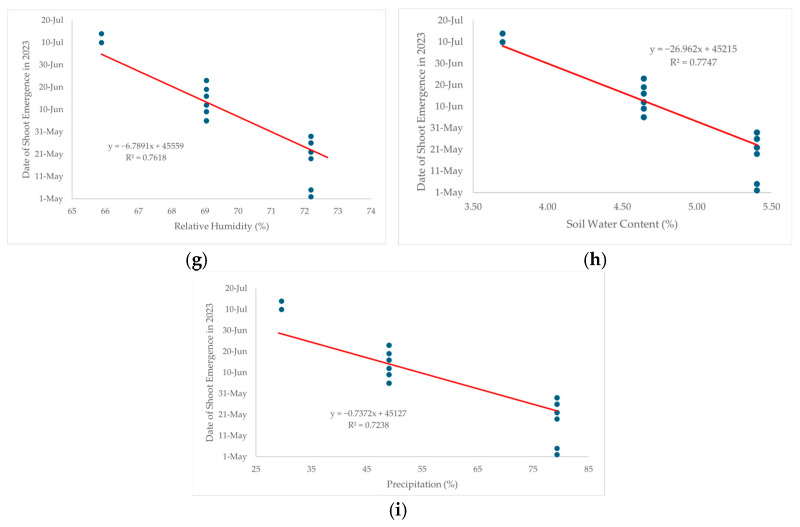
Relationship between bamboo shoot phenology and environmental parameters by regression analysis, relationship between date of shoot emergence in 2022, and air temperature (**a**), soil temperature (**b**), relative humidity (**c**), soil water content (**d**), relationship between date of shoot emergence in 2023 and air temperature (**e**), soil temperature (**f**), relative humidity (**g**), soil water content (**h**), and precipitation (**i**).

Regression analysis was carried out to observe the relationship between phenological factors and environmental parameters. The regression analysis showed significant relationships between shoot phenology and weather variables in 2022, as well as in 2023 (Figure 5). In the year 2022, the date of shoot emergence was strongly influenced by air temperature (r^2^ = 0.8191, coefficient = 1.371, *p* < 0.005) (Figure 5a) and by soil temperature (r^2^ = 0.956, coefficient = 5.616, *p* < 0.001) (Figure 5b), which means that if the air temperature and soil temperature increased by 1 °C, the shooting date was likely to be delayed. During this period, 32 bamboo taxa were observed to produce new shoots emerging in May, 8 taxa in June, and 2 taxa in July. The relationship between date of shoot emergence and temperature was very strong, as indicated by the adjusted r values of 0.773 and 0.815. However, a strong negative relationship was found between the date of shoot emergence and relative humidity (r^2^ = 0.819, coefficient = −0.5997, *p* < 0.001, r = 0.673) (Figure 5c). A similar negative relationship was observed with soil water content (r^2^ = 0.809, coefficient = −17.205, *p* < 0.001, r = 0.805) (Figure 5d). When the relative humidity was low, the bamboo shoot sprouting date was delayed. In our study, the relative humidity was higher in May compared to June and July, resulting in a greater number of bamboo species with new shoots emerging in May. Additionally, the soil water content was also higher in May than in the following two months, further facilitating the early sprouting of most bamboo species during that time.

In the year 2023, a significant relationship was observed between the date of shoot emergence and air temperature (r^2^ = 0.781, coefficient = 0.768, *p* < 0.001) (Figure 5e). A similar close relationship was found with the soil temperature (r^2^ = 0.781, coefficient = 0.235, *p* < 0.001) (Figure 5f). Our analysis indicated that as the temperature increased, the timing of the shoot emergence was delayed. In the year 2023, during the shooting period from May to July, 23 taxa were observed to produce new shoots in May and 17 taxa in June, while 2 taxa exhibited a late shoot emergence in July. Additionally, three environmental factors, i.e., relative humidity, soil water content, and precipitation, were found to influence shoot emergence in *Phyllostachys* taxa. A negative relationship was identified between the date of shoot emergence and relative humidity (r^2^ = 0.762, coefficient = −6.789, *p* < 0.001) (Figure 5g). Similarly, negative relationships were observed with soil water content (r^2^ = 0.775, coefficient = −26.962, *p* < 0.001) (Figure 5h) and precipitation (r^2^ = 0.724, coefficient = −0.737, *p* < 0.001) (Figure 5i). These findings suggest that decreased precipitation, reduced relative humidity, and lower soil water content contribute to delayed shoot emergence in *Phyllostachys* taxa.

Comparing the two years of observation, our study demonstrated that temperature significantly influenced the date of shoot emergence. Correlation analyses revealed the relationship between air temperature and date of shoot emergence, a finding further supported by regression analysis. In 2022, a strong relationship was observed (r^2^ = 0.819) when the air temperature reached between 17–24 °C. Similarly, air temperature also affected shoot emergence in 2023 (r^2^ = 0.781) when the air temperature reached 15.8–22.8 °C. Notably, in March and April 2022, the air temperatures were relatively low (5.8 °C and 17.4 °C); however, there was a sudden increase of 8 °C, from 9.4 °C (April) to 17.4 °C (May). This abrupt rise, along with a 4 °C difference in temperatures in March and April, contributed to earlier shoot emergence in 2022. In contrast, the temperature difference in March and April 2023 was only 2 °C, with a subsequent increase to 15.8 °C in May, i.e., a 6 °C difference between April and May. The temperature fluctuations in 2023 were not as pronounced as those in 2022, indicating that sharp changes in air temperature are crucial in determining the shooting time of *Phyllostachys* spp.

## 3. Discussion

In our study, the observed shoot phenological parameters of the *Phyllostachys* taxa also showed that the individual taxa consistently differed from one another, as was reported earlier in the literature [17,22]. Shoot emergences were observed in May, June, and July, with most species starting to shoot in May, followed by June, and only a few in July, which is generally similar to what is reported in the literature [17,22]. The only European phenological observation of several *Phyllostachys* taxa was reported from Italy [24]. *P. viridiglaucescens*, *P. edulis*, and *P. bambusoides* were reported to have their shooting periods in April and May in Rome, Italy [24]; however, in our study, these species began new shoot emergence later, in May and June. Though local climatic and yearly weather conditions affect the shooting period of *Phyllostachys* taxa [17,25], it was still interesting to compare our results with those reported in the literature, primarily from China.

According to the botanical literature, *Phyllostachys dulcis* produces new shoots in late April in Jiangsu and Zhejiang, China [22]. The shooting period of *P. dulcis* was also studied in Linan, China [19]. The shooting period was from 15 April to 6 May in the first observation year and 17 April to 9 May in the second. The total shooting period was 22 and 23 days, respectively. In our study, shooting was observed to occur more than one month later, from 23 May and 12 June in 2022 and 2023, respectively.

In China, based on the botanical literature, *P. edulis* is said to form its new shoots in May [22], but in field experiments, it began to sprout in the middle of March to early April in China [16], while in another report, it sprouted from 1st of December to the last week of February on the banks of the middle and lower reaches of the Yongbu River, China [26]. The shooting period of *P. pubescens* (*P. edulis*) in Hunan Province, China, was from the last week of March to the middle ten days of April [27], and in Haiziping, Yunnan Province, China, from the middle of March to May [28]. The shooting period of *P. heterocycla* (*P. edulis*) was also observed in Hunan Province, China [29]; it was from the middle of March to the middle of April, and in another report, also from Hunan Province [30], its shooting period was from mid-March to mid-April. According to the findings of those authors, the shoot phenology of this species does not seem to change much from one year to another [29,30]. The above differences in results between the literature and our results, i.e., 23–26 May and 29 May to 9 June during the two observation years, respectively, show that differences in local climatic conditions, namely, in air temperature, influence the phenological changes of plants in general [31].

*P. fimbriligula* generally produces its new shoots in May in Zhejiang and Anhui, China [22]. The shooting period of *P. fimbriligula* was reported to be from 10 April to 31 May in the lower and middle areas of the Yongbu River, China [21]. Also, the shooting period lasted from 2–17 May in a forest near Hangzhou, China [32]. The shooting period was also studied in Linan, China; it was from 15–30 April and 19 April to 3 May in two consecutive years [19]. In our observations, the shooting period began in 22–23 May, which is in the late period reported in the literature.

*P. glauca* usually grows its new shoots from the middle of April to late May in China [22]. Shoot emergence was reported to begin in early or the middle of April, and continued for 25 days in Luoning Country, Henan Province [33]. The shooting period was also studied for three consecutive years in Donggang District, China [23]. Shoot emergence started in late April, and the shooting period was 40–70 days long [23]. According to our results, the shooting period was from 16 May to 6 June and 26 May to 23 June in the two observation years, respectively, which is about one month later compared to the literature.

*P. iridescens* generally produces new shoots in mid- to late April in China [22]. The shooting period was reported to be from 6 April to 18 May and 8 April to 16 May in two consecutive years in Linan, China, [19]. The shooting period was also observed to begin from 19 March to 30 April in a forest near Hangzhou, China [32]. Our observations showed that its shooting period started from 16 May and 26 May in the two study years, which is later than what is reported in the literature.

The emergence of new shoots in *P. mannii* usually starts in early May in China [22]. Its shoot phenology was reported from the Southwest Forestry University, Yunnan, China, where it started shooting in March [34]. We report a much later shooting period, i.e., from 26 May to 9 June and 5–16 June.

In China, *P. meyeri* generally produces new shoots in late April [22]. Its shooting time was found to begin in the first week of May and lasted for 16 days at Dixi Town, Wuxing District, Huzhou City, China [35]. In our observations, it started shooting one month later compared to the literature, on 9 June and 5 June.

In China, *P. nigella* usually grows new shoots in May [22]. The shooting period was reported from 15 April to 9 May and 16 April to 9 May in Linan, China [19]. Our results indicated shooting periods from 30 May to 9 June and 5–9 June, which is later than what is reported in the literature.

In China, the new shoots of *P. nigra* generally grow in late April [22]. Shoot emergence was reported from 21 April to 17 May in Anji, Zhejiang, China [36]. Four cultivars of *P. nigra* were examined in Zhejiang, China [21]. A cultivar named LBH began shoot growth on 5 April, with the other three cultivars starting shooting on 13 April. Their shooting periods were reported to last 20 to 30 days in total. Also, in Taiping, Anhui Province, China, the shooting period was from 23 April to 10 May [18]. The findings of those authors were similar to those in [19], i.e., indicating that daily temperature fluctuations also influence phenology. The shooting period of *P. nigra* var. *henonis* was also observed in Jiangxi Province, China [37]. The shooting period was from mid–late April to early May during 3 consecutive years and lasted 30–35 days total. In our observation, the shooting period was later, between 26 May to 20 June.

In China, *P. prominens* usually grows its new shoots in May [22]. The shooting period of *P. prominens* was studied in Linan, China [19]. The shooting period was between 9–24 April and 12–27 April in the two observation years. Our results showed that the shooting period was later, i.e., in 9 May to 9 June and 5 June to 16 June.

In China, *P. sulphurea* shoots generally emerge in the middle of May [22]. The shooting period was studied in Linan, China [19]; it was reported to be from 6 May to 1 June and from 8–31 May in the two years. Our research revealed a shooting period more than a month later, from 10–21 July.

The new shoots of *P. viridiglaucescens* usually grow in late April in China [22,38]. The shooting period of this species was reported from Linan, China [19]; it was from 5 April to 5 May. Our data indicate that the shooting period was later, i.e., from 9 May to 16 June and 26 May to 16 June.

The new shoots of *P. vivax* and its forms generally grow from mid- to late April in China [26,39]. *P. vivax* f. *aureocaulis* shoots started to grow from 8 May to 12 June in Wangsu Province, China [39]. In Anji, Zhejiang Province, China it began to shoot from 23 April to 26 May [40]. The shooting period of *P. vivax* f. *huangwenzhu* was from late March to late June in Yangzhou, Jiangsu, China [41] and from the end of March to the end of June in Lishui, Zhejiang province, China [42]. In our study, we observed a later shooting period, i.e., from 23 May to 9 June and 5–12 June.

A report similar to our two-year detailed phenological observations of *Phyllostachys* taxa has not been recorded in the scientific literature for Central European climatic conditions. Also, specific phenological observations reported in our study for 11 *Phyllostachys* taxa, namely, *P. aureosulcata* f. *harbin inversa*, *P. bambusoides* f. *holochrysa*, *P. bambusiodes* f. *tanakae*, *P. humilis*, *P. nigra* f*. boryana*, *P. vivax* f. *huangwenzhu inversa*, *P. hispida*, *P. lithophila*, *P. rutila*, *P. virella*, and *P. yunhoensis*, have not previously been reported in such detail along with environmental data in the scientific literature. Rather, to date, only generalized monthly statements without specific geographic, climatic, or weather data are available in the botanical literature [22,38,43].

The effect of soil temperature on the date of shoot emergence was also observed in our study. Most bamboo genotypes ended their shooting period in June, when the soil temperature reached 18.6 °C in 2022 and 16.3 °C in 2023. Only two taxa in 2022 and three taxa in 2023 extended their shooting period into early July, when soil temperatures were 23.3 °C and 20.8 °C, respectively. A related study on the effects of soil temperature on various *Phyllostachys* spp. in China indicated that shoot emergence began at soil temperatures of 8–8.5 °C, with shoot numbers increasing as soil temperatures rose to between 10 °C and 16 °C and ceasing when temperatures reached 16 °C [18]. Our study observed a similar pattern, with soil temperatures of 8.1 °C in April 2022 and 8.6 °C in April 2023, followed by increases to 14.1 °C in May 2022 and 12.4 °C in May 2023. These critical soil temperature thresholds are essential for triggering the new shoot sprouting. If the soil temperature does not reach these critical levels, shoot emergence might be delayed. In our study region, soil temperatures typically reached the necessary threshold between April and May, leading to the emergence of new shoots in May, despite some literature suggesting that the main shooting period for most *Phyllosatchys* taxa occurs in March and April [22,38,43].

## 4. Materials and Methods

### 4.1. Experimental Site and Bamboo Species

Our observations were conducted at the bamboo collection of the Gödöllő Botanical Garden of the Hungarian University of Agriculture and Life Sciences (MATE), in the town of Gödöllő, Hungary, located at 47°35′34″ N 19°22′01″ E, where 42 bamboo genotypes (forms, varieties) of 32 *Phyllostachys* species are maintained under natural conditions. Three millimeters of water was applied to all taxa by drip irrigation during the whole growing period. The names of the *Phyllostachys* taxa used in this study are shown in Table 7.

### 4.2. Data Collection

Different shoot phenological data for *Phyllostachys* taxa, namely, the starting date of shoot emergence, the last date of shoot emergence, the shooting period, the total number of shoots, the number of aborted shoots, and the survival rate of shoots, were collected in the growing period of 2022 and 2023. The environmental data, i.e., the average air temperature, maximum and minimum air temperatures, relative humidity, precipitation, soil temperature at 50 cm depth, and soil water content at 50 cm depth, were measured on site at MATE, Gödöllő, to study the relationship with the shoot phenology of *Phyllostachys* taxa. Phenological data collection was carried out two days per week during the growing seasons from May to August.

### 4.3. Data Analysis

SPSS version 29 and Microsoft excel were used for statistical analyses. Descriptive statistics for phenological data were analyzed to compare the differences of shoot phenology among *Phyllostachys* genotypes. Correlation analyses between the temperature and phenological data were carried out to identify the relationship between shoot the phenology of *Phyllostachys* genotypes and temperature. The influence of environmental parameters on bamboo phenology was studied with the help of linear regression analysis.

## 5. Conclusions

Determining the shoot phenology of *Phyllostachys* taxa is important for our understanding of temperate bamboo adaptability in non-native areas of the world with different climatic conditions. It could also be a tool for monitoring climate change in the long term. This study aimed to study the shoot phenological changes of the temperate bamboo genus *Phyllostachys* spp. grown under the Central European climatic conditions of Hungary. Two years of data on shoot phenology were analyzed with environmental variables. The shooting period was observed to be between May and July, and most species started shooting in May, followed by June. The shoot emergence was earlier in 2022 than in 2023 and lasted from 3–27 days. Bamboo shoot phenology was strongly influenced by air temperature and soil temperature each year. According to the regression analysis, the date of shoot emergence was influenced by air temperature (r^2^ = 0.819) in 2022 and (r^2^ = 0.781) in 2023, and soil temperature also influenced the shoot emergence (r^2^ = 0.967) in 2022, and (r^2^ = 0.781) in 2023. The soil temperature increased to 14.1 °C during shooting time in May. The abrupt change in air temperature between the shooting period and the month prior to shoot initiation determined the early or late shooting date. The soil temperature had to reach 9 °C or above prior to the shooting period, which was found to be a critical temperature for shoot emergence of *Phyllostachys* bamboo taxa. Our results indicate similar shooting order for the taxa as in the literature, except our data show up to one month later shooting periods for all taxa compared to that reported in the literature. The shooting phenology for eleven *Phyllostachys* taxa, namely *P. aureosulcata* f. *harbin inversa*, *P. bambusoides* f. *holochrysa*, *P. bambusiodes* f. *tanakae*, *P. humilis*, *P. nigra* f*. boryana*, *P. vivax* f. *huangwenzhu inversa*, *P. hispida*, *P. lithophila*, *P. rutila*, *P. virella*, and *P. yunhoensis,* had not been previously reported in such detail, along with environmental data, in the scientific literature.

## Figures and Tables

**Table 1:** Data analysis of shoot phenology data collected in 2022.

Genotype	DOE	EOS	TOS	NOL	NOD	SR	SHP
*P. acuta*	19-May	02-Jun	7	5	2	71.43	14
*P. angusta*	26-May	13-Jun	30	14	16	46.67	18
*P. arcana* f. *luteosulcata*	09-May	02-Jun	27	18	9	66.67	24
*P. aureosulcata*	09-May	02-Jun	51	20	31	39.22	24
*P. aureosulcata* f. *harbin inversa*	02-May	02-Jun	17	17	0	100.00	31
*P. aureosulcata* f. *pekinensis*	09-May	19-May	16	10	6	62.50	10
*P. aureosulcata* f. *aureocaulis*	16-May	30-May	23	12	11	52.17	14
*P. aureosulcata* f. *spectabilis*	12-May	26-May	18	9	9	50.00	14
*P. bambusoides* f. *holochrysa*	13-Jun	30-Jun	7	6	1	85.71	17
*P. bambusoides* f. *tanakae*	13-Jun	23-Jun	15	12	3	80.00	10
*P. dulcis*	23-May	23-May	2	2	0	100.00	1
*P. edulis*	23-May	26-May	6	6	0	100.00	3
*P. flexuosa*	12-May	26-May	18	8	12	44.44	14
*P. humilis*	16-May	30-May	9	3	6	33.33	14
*P. iridescens*	16-May	16-May	1	1	0	100.00	1
*P. meyeri*	09-Jun	09-Jun	3	3	0	100.00	1
*P. nigra* f. *boryana*	19-May	13-Jun	18	8	10	44.44	25
*P. nigra* var. *nigra*	26-May	20-Jun	32	12	22	37.50	25
*P. platyglossa*	16-May	30-May	12	10	2	83.33	14
*P. vivax*	23-May	02-Jun	29	17	12	58.62	10
*P. vivax* f. *aureocaulis*	23-May	09-Jun	15	8	7	53.33	17
*P. vivax* f. *huangwenzhu*	09-Jun	09-Jun	1	1	0	100.00	1
*P. vivax* f. *huangwenzhu-inversa*	26-May	09-Jun	8	7	1	87.50	14
*P. bissettii*	09-May	19-May	21	16	5	76.19	10
*P. circumpilis*	23-May	09-Jun	7	7	0	100.00	17
*P. fimbriligula*	23-May	23-May	1	1	0	100.00	1
*P. mannii*	26-May	09-Jun	18	8	10	44.44	14
*P. glauca*	16-May	06-Jun	30	24	6	80.00	21
*P. heteroclada*	12-May	02-Jun	27	19	8	70.37	21
*P. hispida*	16-May	02-Jun	10	2	8	20.00	17
*P. lithophila*	13-Jun	27-Jun	4	3	1	75.00	14
*P. rubromarginata*	02-Jun	23-Jun	60	22	38	36.67	21
*P. nigella*	30-May	09-Jun	15	8	7	53.33	10
*P. prominens*	09-May	09-Jun	7	6	1	85.71	31
*P. rutila*	16-May	06-Jun	42	22	20	52.38	21
*P. glabrata*	12-May	09-Jun	48	14	34	29.17	28
*P. virella*	09-Jun	23-Jun	29	21	8	72.41	14
*P. viridiglaucescens*	09-May	16-Jun	57	22	35	38.60	38
*P. sulphurea* f. *houzeauana*	09-Jun	09-Jun	1	1	0	100.00	1
*P. yunhoensis*	23-May	23-Jun	53	20	33	37.74	31
*P. sulphurea* f. *robert young*	04-Jul	04-Jul	2	2	0	100.00	1
*P. sulphurea* f. *viridis*	04-Jul	04-Jul	2	2	0	100.00	1
Std Error	2.24	2.24	2.55	1.11	1.69	3.89	1.70
Variance	210.58	210.74	274.12	51.44	119.50	633.34	122.33
*p* value	**	**	**	**	**	**	**

DOE—date of first shoot emergence, EOS—date of last shoot emergence, TOS—total number of shoots, NOL—number of surviving shoots, NOD—number of aborted shoots, SR—shoot survival rate, SHP—shooting period (days). ** = *p* < 0.001.

**Table 2:** The data analysis of shoot phenology collected in 2023.

Genotype	DOE	EOS	TOS	NOL	NOD	SR	SHP
*P. acuta*	22-May	05-Jun	26	5	21	19.23	14
*P. angusta*	22-May	16-Jun	24	7	17	29.17	25
*P. arcana* f. *luteosulcata*	05-May	26-May	19	8	11	42.11	21
*P. aureosulcata*	19-May	29-May	35	12	23	34.29	10
*P. aureosulcata* f. *harbin inversa*	02-May	22-May	35	11	24	31.43	20
*P. aureosulcata* f. *pekinensis*	22-May	29-May	35	7	28	20.00	7
*P. aureosulcata* f. *aureocaulis*	29-May	09-Jun	9	7	2	77.78	11
*P. aureosulcata* f. *spectabilis*	29-May	09-Jun	2	1	1	50.00	11
*P. bambusoides* f. *holochrysa*	23-Jun	26-Jun	2	2	0	100.00	3
*P. bambusoides* f. *tanakae*	16-Jun	26-Jun	14	4	10	28.57	10
*P. dulcis*	12-Jun	12-Jun	2	2	0	100.00	1
*P. edulis*	29-May	09-Jun	6	6	0	100.00	11
*P. flexuosa*	22-May	05-Jun	63	22	41	34.92	14
*P. humilis*	26-May	05-Jun	29	10	19	34.48	10
*P. iridescens*	26-May	05-Jun	12	3	9	25.00	10
*P. meyeri*	05-Jun	09-Jun	6	5	1	83.33	4
*P. nigra* f. *boryana*	29-May	23-Jun	4	2	2	50.00	25
*P. nigra* var. *nigra*	12-Jun	12-Jun	1	1	0	100.00	1
*P. platyglossa*	29-May	16-Jun	16	11	5	68.75	18
*P. vivax*	29-May	12-Jun	29	11	18	37.93	14
*P. vivax* f. *aureocaulis*	05-Jun	12-Jun	5	2	3	40.00	7
*P. vivax* f. *huangwenzhu*	19-Jun	19-Jun	1	1	0	100.00	1
*P. vivax* f. *huangwenzhu inversa*	05-Jun	16-Jun	12	4	8	33.33	11
*P. bissettii*	22-May	05-Jun	22	10	12	45.45	14
*P. circumpilis*	09-Jun	23-Jun	5	5	0	100.00	14
*P. fimbriligula*	22-May	26-May	4	2	2	50.00	4
*P. mannii*	05-Jun	16-Jun	4	4	0	100.00	11
*P. glauca*	26-May	23-Jun	31	18	13	58.06	28
*P. heteroclada*	26-May	05-Jun	14	7	7	50.00	10
*P. hispida*	29-May	05-Jun	7	7	0	100.00	7
*P. lithophila*	23-Jun	10-Jul	2	2	0	100.00	17
*P. rubromarginata*	09-Jun	26-Jun	23	13	10	56.52	17
*P. nigella*	05-Jun	12-Jun	7	4	3	57.14	7
*P. prominens*	05-Jun	16-Jun	10	7	3	70.00	11
*P. rutila*	26-May	12-Jun	45	15	30	33.33	17
*P. glabrata*	26-May	12-Jun	30	6	24	20.00	17
*P. virella*	16-Jun	23-Jun	6	5	1	83.33	7
*P. viridiglaucescens*	26-May	16-Jun	6	3	3	50.00	21
*P. sulphurea* f. *houzeauana*	14-Jul	14-Jul	1	1	0	100.00	1
*P. yunhoensis*	05-Jun	12-Jun	9	3	6	33.33	7
*P. sulphurea* f. *robert young*	10-Jul	21-Jul	5	5	0	100.00	11
*P. sulphurea* f. *viridis*	23-Jun	23-Jun	1	1	0	100.00	1
Std Error	2.24	1.91	2.19	0.74	1.59	4.55	1.07
Variance	211.65	153.49	202.39	23.16	106.5	868.85	48.20
*p* value	**	**	**	**	**	**	**

DOE—date of first shoot emergence, EOS—date of last shoot emergence, TOS—total number of shoots, NOL—number of surviving shoots, NOD—number of aborted shoots, SR—shoot survival rate, SHP—shooting period (days). ** = *p* < 0.001.

**Table 3:** Descriptive statistics of collected data from the two years of observation.

	Year	Mean	Minimum	Maximum	StD	CV%
Date of first shoot emergence	2022	44,704.90	44,683.00	44,746.00	14.51	0.03
Number of shoots		19.02	1.00	60.00	16.56	87.03
Date of last shoot emergence		44,720.43	44,697.00	44,774.00	14.52	0.03
Number of surviving shoots		10.21	1.00	24.00	7.17	70.22
Number of aborted shoots		8.90	0.00	38.00	10.93	122.76
Survival rate		68.31	20.00	100.00	25.17	36.84
Shooting period (Days)		15.69	1.00	49.00	11.02	70.09
Date of shoot emergence	2023	45,079.64	45,048.00	45,121.00	14.55	0.03
Number of shoots		14.74	1.00	63.00	14.23	96.53
Date of last shoot emergence		45,090.98	45,068.00	45,128.00	12.39	0.03
Number of surviving shoots		6.24	1.00	22.00	4.81	77.15
Number of aborted shoots		8.50	0.00	41.00	10.32	121.41
Survival rate		60.65	19.23	100.00	29.48	48.60
Shooting period (Days)		11.40	1.00	28.00	6.94	60.75

**Table 4:** Environmental parameters collected before the taking of observations in 2022 and 2023.

	2022		2023	
	March	April	March	April
Average Air temperature (°C)	5.88	9.41	7.63	9.58
Average Maximum Air Temperature (°C)	21.7	20.6	22.5	21.8
Average Minimum Air Temperature(°C)	−6.5	−3.3	−3.2	−2.3
Soil Temperature at 50 cm depth (°C)	3.76	8.15	5.72	8.66
Soil Water Content at 50 cm depth (%)	4.84	4.91	7.52	6.44
Precipitation (mm)	10.4	45.5	33.0	28.4
Relative Humidity (%)	49.14	65.68	64.17	67.98

**Table 5:** Environmental parameters collected during the observations in 2022 and 2023.

Parameters	May		June		July	
	2022	2023	2022	2023	2022	2023
Average Air Temperature (°C)	17.40	15.86	22.26	19.83	24.03	22.83
Average Maximum Air Temperature (°C)	22.74	20.42	28.17	24.83	30.12	28.31
Average Minimum Air Temperature(°C)	11.89	11.36	16.01	14.64	17.39	17.26
Soil Temperature at 50 cm depth (°C)	14.095	12.44	18.65	16.35	23.30	20.81
Soil Water Content at 50 cm depth (%)	4.47	5.39	2.97	4.62	1.94	3.67
Precipitation (mm)	29.5	79.3	41.1	49	25	29.6
Relative Humidity (%)	61.28	72.25	58.90	69.11	49.14	65.97

**Table 6:** Correlation analysis of bamboo phenology and air temperature and soil temperature; (**a**) 2022, (**b**) 2023.

**(a)**					
	**DOE22**	**TOS22**	**NOL22**	**SR22**	**SHP22**
Average Air temperature (°C)	0.883 **	−0.247	−0.255	0.393 *	−0.259
Average Maximum Air Temperature (°C)	0.882 **	−0.247	−0.255	0.393 *	−0.259
Average Minimum Air Temperature (°C)	0.881 **	−0.246	−0.254	0.392 *	−0.257
Soil Temperature at 50 cm depth (°C)	0.978 **	−0.381 *	−0.436 **	0.353 *	−0.422 **
Soil Water Content at 50 cm depth (%)	−0.998 **	0.367 *	0.400 **	−0.404 **	0.373 *
Precipitation (mm)	0.413 **	−0.067	−0.052	0.184	−0.018
Relative Humidity (%)	0.0005	0.114	0.128	0.055	−0.051
**(b)**					
	**DOE23**	**TOS23**	**NOL23**	**SR23**	**SHP23**
Average Air temperature (°C)	0.569 **	−0.449 **	−0.424 **	0.381 *	−0.509 **
Average Maximum Air Temperature (°C)	0.575 **	−0.446 **	−0.421 **	0.378 *	−0.507 **
Average Minimum Air Temperature (°C)	0.577 **	−0.445 **	−0.420 **	0.377 *	−0.506 **
Soil Temperature at 50 cm depth (°C)	0.079	−0.582 **	−0.471 **	0.670 **	−0.472 **
Soil Water Content at 50 cm depth (%)	−0.071	0.590 **	0.480 **	−0.628 **	0.507 **
Precipitation (mm)	−0.284	−0.051	−0.085	−0.015	0.047
Relative Humidity (%)	0.077	0.094	0.192	0.045	0.161

DOE—date of shoot emergence, TOS—total number of shoots, NOL—number of surviving shoots, SR—survival rate (%), SHP—shooting period. **—*p* < 0.001, *—*p* < 0.05.

**Table 7:** The names of *Phyllostachys* taxa used in this study.

**No.**	**Species**	**No.**	**Species**
1	*P*. *acuta*	22	*P. vivax* f. *aureocaulis*
2	*P. angusta*	23	*P. vivax* f. *huangwenzhu*
3	*P. arcana* f. *luteosulcata*	24	*P. vivax* f. *huangwenzhu inversa*
4	*P. aureosulcata*	25	*P. bissettii*
5	*P. aureosulcata* f. *harbin inversa*	26	*P. circumpilis*
6	*P. aureosulcata* f. *pekinensis*	27	*P. fimbriligula*
7	*P. aureosulcata* f. *aureocaulis*	28	*P. mannii*
8	*P. aureosulcata* f. *spectabilis*	29	*P. glauca*
9	*P. bambusoides* f. *holochrysa*	30	*P. heteroclada*
10	*P. bambusoides* f. *tanakae*	31	*P. hispida*
11	*P. dulcis*	32	*P. lithophila*
12	*P. edulis*	33	*P. rubromarginata*
13	*P. flexuosa*	34	*P. nigella*
14	*P. humilis*	35	*P. prominens*
15	*P. iridescens*	36	*P. rutila*
16	*P. meyeri*	37	*P. glabrata*
17	*P. nigra* f. *boryana*	38	*P. virella*
18	*P. nigra* var. *nigra*	39	*P. viridiglaucescens*
19	*P. platyglossa*	40	*P. sulphurea* f. *houzeauana*
20	*P. sulphurea* f. *viridis*	41	*P. yunhoensis*
21	*P. vivax*	42	*P. sulphurea* f. *robert young*

## Data Availability

The data will be provided on reasonable request from the corresponding author.
